# Molecular Analysis of Fungal Populations in Patients with Oral Candidiasis Using Internal Transcribed Spacer Region

**DOI:** 10.1371/journal.pone.0101156

**Published:** 2014-06-30

**Authors:** Shinsuke Ieda, Masafumi Moriyama, Toru Takashita, Takashi Maehara, Yumi Imabayashi, Shoichi Shinozaki, Akihiko Tanaka, Jun-Nosuke Hayashida, Sachiko Furukawa, Miho Ohta, Yoshihisa Yamashita, Seiji Nakamura

**Affiliations:** 1 Section of Oral and Maxillofacial Oncology, Division of Maxillofacial Diagnostic and Surgical Sciences, Faculty of Dental Science, Kyushu University, Fukuoka, Japan; 2 Section of Preventive and Public Health Dentistry, Division of Oral Health, Growth and Development, Faculty of Dental Science, Kyushu University, Fukuoka, Japan; Louisiana State University, United States of America

## Abstract

Oral candidiasis is closely associated with changes in the oral fungal flora and is caused primarily by *Candida albicans*. Conventional methods of fungal culture are time-consuming and not always conclusive. However, molecular genetic analysis of internal transcribed spacer (ITS) regions of fungal rRNA is rapid, reproducible and simple to perform. In this study we examined the fungal flora in patients with oral candidiasis and investigated changes in the flora after antifungal treatment using length heterogeneity-polymerization chain reaction (LH-PCR) analysis of ITS regions. Fifty-two patients with pseudomembranous oral candidiasis (POC) and 30 healthy controls were included in the study. Fungal DNA from oral rinse was examined for fungal species diversity by LH-PCR. Fungal populations were quantified by real-time PCR and previously-unidentified signals were confirmed by nucleotide sequencing. Relationships between the oral fungal flora and treatment-resistant factors were also examined. POC patients showed significantly more fungal species and a greater density of fungi than control individuals. Sixteen fungi were newly identified. The fungal populations from both groups were composed predominantly of *C. albicans*, though the ratio of *C. dubliniensis* was significantly higher in POC patients than in controls. The diversity and density of fungi were significantly reduced after treatment. Furthermore, fungal diversity and the proportion of *C. dubliniensis* were positively correlated with treatment duration. These results suggest that *C. dubliniensis* and high fungal flora diversity might be involved in the pathogenesis of oral candidiasis. We therefore conclude that LH-PCR is a useful technique for diagnosing and assessing the severity of oral candidal infection.

## Introduction

In recent days, interest in oral hygiene in elderly and immunocompromised individuals is increasing because of the associations between oral candidiasis and increased morbidity and mortality from aspiration pneumonia in these patients [1], [2]. Although systemic candidiasis caused by *Candida albicans* is an opportunistic infection of the oral mucosa, the widespread use of empiric and prophylactic antifungal drugs during the last 20 years has caused a shift in fungal flora towards other yeast species such as *C. glabrata, C. tropicalis, C. krusei, C. dubliniensis* and non-*Candida* species [3]. More recently, invasive infections caused by yeasts with intrinsic or acquired resistance to antifungal drugs have become a major public health problem [4], [5]. It has been reported that the delayed initiation of antifungal drugs for candidal infection leads to poor clinical outcomes and increased mortality [6], [7] which has underlined the necessity for early identification and appropriate antifungal treatment. However, the conventional identification kits for oral fungi are based on cultivation methods which are often time consuming and not always conclusive [8]. In contrast, recent genetic approaches using length heterogeneity-polymerase chain reaction (LH-PCR) analysis of internal transcribed spacer (ITS) regions of fungal nuclear rRNA have enabled more exhaustive surveys of fungal communities including the difficult-to-cultivate species [9], [10]. The ITS1-5.8S rRNA-ITS2 region is the most widely-sequenced DNA region in fungal species, and can be rapidly amplified using universal fungal primers. These amplicons provide phylogenetic sequence information, especially at lower taxonomic levels [11].

However, this technique has not yet been applied to serial clinical assessments including the effects of treatment on fungal flora. We therefore examined the diversity of fungal species in patients with pseudomembranous oral candidiasis (POC) by LH-PCR, and identified the factors associated with resistance to antifungal therapy.

## Materials and Methods

### Ethics Statement

The study design was approved by the Ethics Committee of Kyushu University, Japan, and all participants provided written informed consent (IRB number: 25-269).

### Patients

This study included 52 patients (5 men and 47 women; mean age ± standard deviation (SD), 62.7±13.4 years) with POC who were referred to the Department of Oral and Maxillofacial Surgery, Kyushu University Hospital, Japan, between 2010 and 2013. These patients had a history of Sjögren’s syndrome (n = 10), hypertension (n = 6), rheumatoid arthritis (RA) (n = 2), and diabetes mellitus (n = 1). None of the patients received previous therapy with antibiotic, steroid, or immunosuppressive drugs within the last 3 months nor used dentures. After diagnosis by direct microscopy, patients were treated with Florid oral gel (FLO-G) 2% 2.5–5 g (miconazole, Mochida Pharmaceutical Co., Ltd. Tokyo, Japan) applied to the oral mucosa four times a day after meals and before sleep. Treatment duration was defined as the period from the initial drug therapy to the disappearance of both white patches on the oral mucosa and subjective complaints, including burning pain or haphalgesia. The average treatment duration for POC was 24.8±10.8 days. Therapeutic process of Thirty-three of the 52 patients could be assessed over time until complete healing. Patients were excluded from the study if they were pregnant, lactating, or hypersensitive to FLO-G. The control group consisted of thirty individuals (8 men, 22 women, mean age ± SD, 61.6±10.6 years) who were non-smokers, non-denture wearers, and had no clinical signs of oral mucosal disease such as xerostomia or reduced saliva production. These individuals had a history of hypertension (n = 5), hyperlipidemia (n = 0), and diabetes mellitus (n = 3). None of them have received therapy with steroidal, immunosuppressive or antifungal drugs.

### Clinical findings

We evaluated stimulated whole salivary flow rate (SWS), unstimulated whole salivary flow rate (UWS), the presence of white patches on the oral mucosa, and clinical epithelial disorders such as pain or burning, which led to poor nutrition and invasive infection. SWS was determined using the Saxon test [12] and UWS was determined using the spitting method [13]. Oral mucosal symptoms on the tongue, angle of the mouth, and buccal mucosa were evaluated by three experienced dentists. POC was diagnosed when two or more dentists agreed on the diagnosis.

### Sample collection

Subjects were asked to rinse their mouths for 30 s with 5 ml normal saline at every visit prior to measuring SWS. Then tongue scraping samples were collected by scraping from the base to the tip of the anterior 2/3 of the tongue dorsum with a sterile plastic spatula (Muddler; Nihon Dixie, Yokohama, Japan) as described previously [11]. Oral rinses and tongue scraping specimens were collected in sterile plastic tubes and stored at −80°C until analysis. In this study, we selected the samples at the first visit and upon healing.

### DNA extraction

DNA was extracted from each sample as described previously [14]. Oral rinses (5 ml) were collected into sterile plastic tubes and fungi were harvested by centrifugation (8500 rpm for 10 min at 4°C). The pellet was then resuspended in 5 ml phosphate-buffered saline and 3 ml of the suspension was then centrifuged (8500 rpm, 10 min, 4°C). The pellet was resuspended in 1 ml of 0.2% sodium dodecyl sulfate (SDS) in 10 mM Tris-HCl, 1 mM EDTA, pH 8.0 (TE). Two microliters of 10 mM RNase (10 µg/ml) was added and heated at 15°C for 10 min. The sample was then passed through a 79-µm filter (Nippon-Clever Co. Ltd., Aichi, Japan) and fixed in a 13-mm Swinny stainless filter holder (Merck Millipore, Japan). The filtrate was re-suspended in 400 µl of 1% SDS in TE. One microliter of the RNase was added and heated at 37°C for 10 min, and the suspension was then added to a plastic tube containing 0.3 g zirconia-silica beads (bead size 0.1 mm; Biospec Products, Bartlesville, OK, USA) and one tungsten carbide bead (bead size 3 mm; Qiagen). The sample was heated at 55°C for 10 min, followed by violent agitation for 3 min in a cell disruptor (Disruptor Genie; Scientific Industries, Inc., Bohemia, NY, USA). After heating at 70°C for 10 min, 500 µl phenol and 300 µl chloroform were added. The supernatant liquid was then collected by centrifugation (15,000 rpm, 5 min, 4°C) and a further 300 µl chloroform was added. 3 M NaAc was added to 10% volume of the supernatant liquid, together with the same quantity of 2-propanol. The sample was then kept on ice for overnight. After centrifugation at 15,000 rpm for 15 min, the nucleic acids were precipitated with 100% ethanol. Following centrifugation, the DNA was washed with 70% ethanol and resuspended in 30 µl TE buffer (1 mM Tris-HCl, 0.1 mM EDTA, pH 8.0). All the DNA samples were kept frozen at −80°C until analysis.

### Quantitative PCR analysis

Real-time PCR of the fungal ITS1 region was used to quantify fungal populations in the gargle specimens. PCR was performed with 25 µl of PCR mixture containing 50 pmol of each primer, ITS1-F (5-CTT GGT CAT TTA GAG GAA GTA A-3′) [15], ITS2 (5-CGC TGC GTT CTT CAT CG-3′) [16] and 1 ml of sample DNA, using a QuantiFast SYBR Green PCR kit (Qiagen, Hilden, Germany) in a 7500 Real-Time PCR System (Applied Biosystems, Foster City, CA, USA). The cycling conditions were 95°C for 10 min, followed by 60 cycles of 95°C for 3 s and annealing and extension at 65°C for 30 s. Fungal quantities were calculated using the comparative Ct method with the DNA extracted from *C. albicans* ATCC 28366 as a control. They were expressed as colony-forming units (CFU) per gargle specimen sample by converting the amount of DNA from *C. albicans* to CFU of the source broth. *C. albicans* was grown aerobically in CHROMagar (Kanto Chemical Co., Inc) *Candida* broth and genomic DNA was extracted by the same method used for the gargle specimens. The CFU of the *C. albicans* broth was calculated on CHROMagar plates. The copy number of the genomic DNA in 1 ml of sample was then determined from the standard curve without taking into account the amount of human DNA present in the samples. The specificity of the PCR was examined by melting curve analysis.

### LH-PCR analysis

The diversity of fungal species in each sample was evaluated based on the length heterogeneity of the ITS1-5.8S rRNA-ITS2 region. This region was amplified using unlabeled ITS1-F and the universal fungal primer ITS4 (5′-TCC TCC GCT TAT TGA TAT GC-3′) labeled at the 5′ end with 6-carboxyfluorescein [11]. Amplification was performed in a 10 µl reaction mixture containing 1 µl of extracted DNA, 1 U of KOD Plus Ver. 2 polymerase (Toyobo, Osaka, Japan), and 10 pmol of each primer. Amplification conditions were 35 cycles of 94.0°C for 15 s, 57.4°C for 30 s, and 68.0°C for 60 s. The products were purified using a Wizard SV Gel and PCR clean-up system (Promega, Madison, WI, USA). The purified amplicons were mixed with 10 µl of deionized formamide and 0.5 µl of GeneScan 1200 LIZ size standard (Applied Biosystems), denatured, and subjected to capillary electrophoresis using an ABI 3130 Genetic Analyzer (Applied Biosystems). The data were analyzed with GeneMapper Ver. 4.0 (Applied Biosystems). After the exclusion of fragments with a peak area <1% of the total, the LH-PCR profiles were aligned. Fragments with lengths that differed by one base or less were considered identical.

### Direct sequencing

The nucleotide sequences of the ITS1-5.8S rRNA-ITS2 fragments were determined to identify their fungal origins. Each ITS1-5.8S rRNA-ITS2 fragment was re-amplified using unlabeled ITS1-F and ITS4 from the DNA samples of subjects with the highest peak area proportions.

### Cloning and sequencing

DNA fragments were amplified using four unlabeled PCR primers: an extended forward primer: ITS1-F (5′-ACA GTG CCC TTG GTC ATT TAG AGG AAG TAA-3′), a non-extended reverse primer: ITS4 (5′-TCC TCC GCT TAT GC-3′), a non-extended forward primer: ITS1-F (5′-CTT GGT CAT TTA GAG GAA GTA A-3′), and an extended reverse primer: ITS4 (5′-ACA GTG GGT CCT CCG CTT ATG C-3′). The amplified fragments were cloned into the vector pHST-1 (Hetero-Stagger PCR Cloning Kit, BioDynamics Laboratory Inc., Tokyo, Japan [17]). Two PCR primers contained eight or nine extra bases at the 5′ ends, depending on non-proofreading or proofreading thermostable DNA polymerase, while the other two primers had no extra bases. Two PCR reactions were set up to generate two PCR products containing different extra terminal sequences. The recombinant plasmids were purified from a culture derived from a single colony, and the nucleotide sequences of the inserts were determined using pHS forward sequence and reverse primers. After confirmation of their lengths, the sequences were compared with the sequences in the nucleotide collection database using the BLAST nucleotide algorithm (http://blast.ncbi.nlm.nih.gov/Blast.cgi). The sequences were identified as a cultivated fungal species if they showed ≥99% similarity. When no cultivated species with 99% similarity was present, the sequences were assigned to the best hit and the percent identity was shown.

### Statistical analysis

Values are given as the mean ± SD. Group results were compared using unpaired Student’s *t*-test, Fisher’s exact test, and Spearman’s rank correlation. A value of *p*<0.05 was considered significant. All statistical analyses were performed using JMP software (version 8; SAS Institute, Japan).

## Results

### Clinical findings

As shown in Table 1, POC patients included a high proportion of elderly women. The SWS of patients with POC was within the normal range (Saxon’s test, 2.19±1.64 g/2 min) while the UWS was lower than the reference value (spitting method, 0.8±0.7 ml/15 min). POC lesions were frequently recognized in the tongue dorsum (78.8%), angulus oris (23.1%), buccal mucosa (17.3%), and palate (7.7%).

**Table 1:** Patient’s profile and clinical findings.

	POC	Control
Men: women	5∶47	8∶22
Mean age	62.7±13.4**	61.6±10.6
SWS		
Saxon’s test (g/2 min)	2.19±1.64**	5.37±1.43
UWS		
Spitting method (ml/15 min)	0.8±0.7**	2.6±0.9
<Lesion location of oral candidiasis>		
Tounge dorsum	41/52 (78.8%)	0/30 (0.0%)
Buccal mucosa	9/52 (17.3%)	0/30 (0.0%)
Palate	4/52 (7.7%)	0/30 (0.0%)
Angulus oris	12/52 (23.1%)	0/30 (0.0%)

POC, pseudomembranous oral candidiasis; *p<0.05, **p<0.01 (Student’s *t*-test); SWS, stimulated whole salivary flow rate; UWS, unstimulated whole salivary flow rate; PCR, polymerization chain reaction; LH-PCR, length heterogeneity-PCR.

### Quantification of fungal populations

Real-time PCR analysis was performed to quantify the fungal population in oral rinses. As shown in Table 2, the total PCR products from the DNA samples of patients with POC were significantly higher than that of the control group. Furthermore, PCR products were significantly decreased after treatment in the POC patients who received the antifungal drug (FLO-G).

**Table 2:** Quantification and species diversity of fungal populations.

	Control	POC	POC treated by antifungal agent(n = 33)	
	(n = 30)	(n = 52)	Before	After
<Quantification by real-time PCR>				
Relative PCR products	1.0±0.4	4.0±1.9*	3.9±0.8	1.4±0.7
<Detection signals by LH-PCR>				
Total number of signals	33	47	45	36
Number of signals per person	6.6±2.3	7.4±2.3*	7.5±2.0	6.4±2.1*

*p<0.05 (Student’s *t*-test).

### Species diversities of fungal populations in POC patients and controls

**Figure 1:**
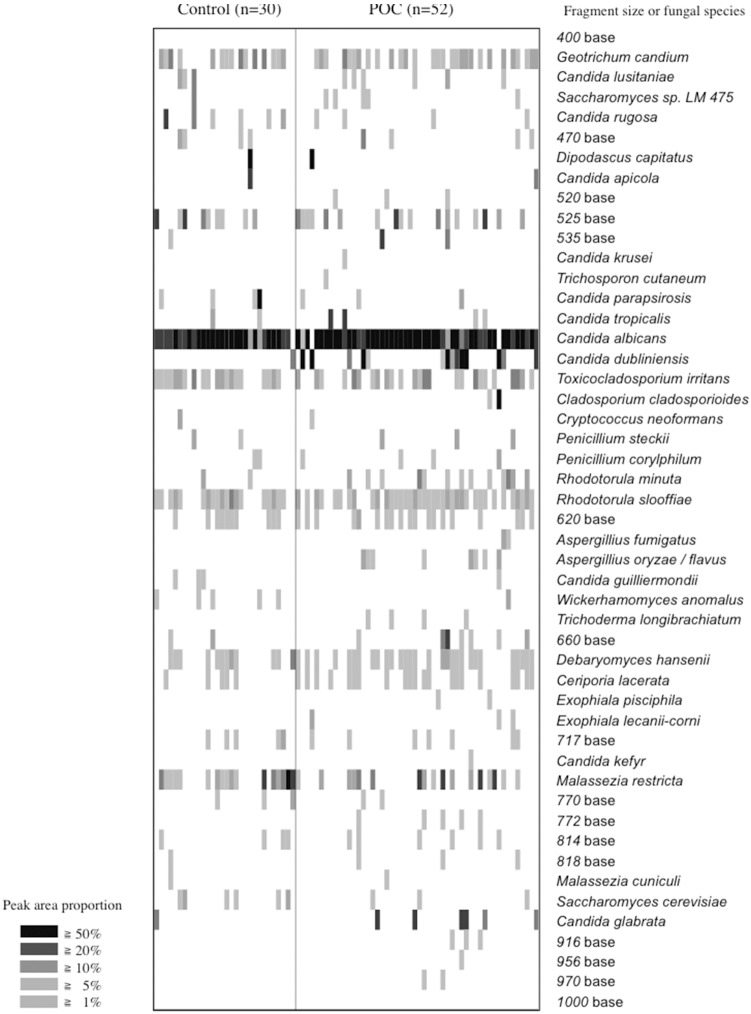
LH-PCR peak patterns in controls and patients with pseudomembranous oral candidiasis (POC) visualized as a gel-like image. The peak area proportion (47 detected peaks) in each LH-PCR profile is represented by the gray-scale intensity of each grid.

**Figure 2:**
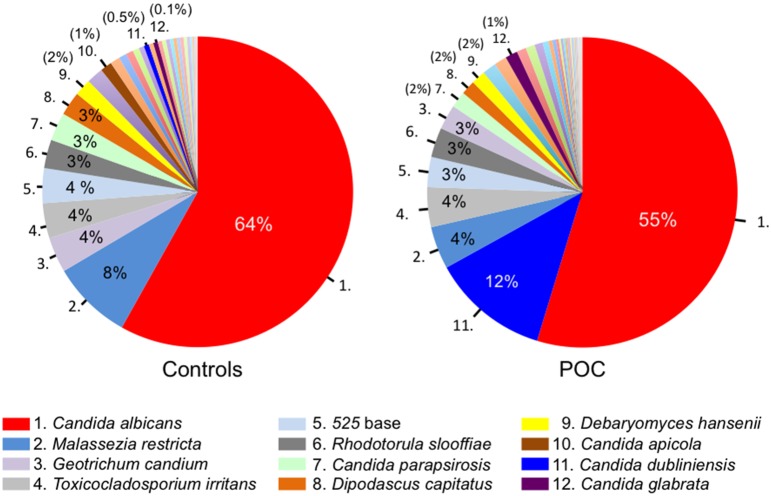
The mean composition ratio of fungal populations from controls and patients with POC. Top 12 fungi of detection rate in control group are shown.

LH-PCR analysis of the ITS1-5.8S rRNA-ITS2 region was conducted to assess the diversity of fungal species. The total and average numbers of detection signals per person were significantly higher in patients with POC than in controls (Table 2). The LH-PCR profiles are displayed as a gel-like image in Figure 1. The overall profiles contained 47 distinct peaks (DNA fragments with different sizes) of 405–970 bases. Thirty-three out of 47 peaks were detected in both controls and patients with POC, whereas 14 in only patients with POC. These fungal origins were identified based on their nucleotide sequences, and the detection ratios of fungal populations from controls and patients with POC were calculated. The fungal origins of the 32 fragments were identified based on their nucleotide sequences. The fragments of 571 and 573 bases that matched *C. dubliniensis* with ≥99% similarity were defined as *C. dubliniensis*. Similarly, the fragments of 763 and 765 bases showed ≥99% similarity with ITS sequences of *Malassezia restricta* and were thus assigned to that species. *C. albicans* (569 bases) was the most frequently-detected fungus in both groups (POC, 96.2%; controls, 96.7%). In contrast, only *C. dubliniensis* was detected at a significantly higher rate in POC patients than in controls (Table 3). The nucleotide sequences of the other detected fragments matched cultivated species with ≥99% similarity. With regard to the composition ratio of fungal populations, *C. albicans* constituted more than half of the total fungal populations in both groups, while *C. dubliniensis* constituted a substantial portion only in the patient group (POC, 12.0%; controls, 0.5%) (Figure 2).

**Table 3:** Fungal species corresponding to each fragment in LH-PCR analysis.

Size (base)	Fungal species corresponding to each fragment	Number of subjects (%)		*P*-value
		POC (n = 52)	Control (n = 30)	
569	*Candida. albicans*	50 (96.2%)	29 (96.7%)	0.558
619	*Rhodotorula slooffiae*	38 (73.1%)	19 (63.3%)	0.416
667	*Debaryomyces hansenii*	29 (55.8%)	12 (40.0%)	0.274
405	*Geotrichum candium*	28 (53.8%)	15 (50.0%)	0.505
578	*Toxicocladosporium irritans*	25 (48.1%)	20 (66.7%)	0.249
620	*620 base*	21 (40.4%)	10 (33.3%)	0.421
669	*Ceriporia lacerata*	19 (36.5%)	5 (16.7%)	0.114
525	*525 base*	17 (32.7%)	9 (30.0%)	0.525
763/765	*Malassezia restricta*	17 (32.7%)	17 (56.7%)	0.129
571/573	*Candida dubliniensis*	14 (26.9%)	1 (3.3%)	0.017*
613	*Rhodotorula minuta*	12 (23.1%)	2 (6.7%)	0.088
717	*717 base*	8 (15.4%)	5 (16.7%)	0.763
625	*Aspergillus oryzae/flavus*	7 (13.5%)		0.050
416	*Candida lusitaniae*	6 (11.5%)	3 (10.0%)	0.579
660	*660 base*	6 (11.5%)	2 (6.7%)	0.408
623	*Aspergillus fumigatus*	6 (11.5%)		0.075
908	*Candida glabrata*	6 (11.5%)	1 (3.3%)	0.226
423	*Saccharomyces sp. LM475*	5 (9.6%)	1 (3.3%)	0.306
814	*814 base*	5 (9.6%)	4 (13.3%)	0.451
552	*Candida parapsirosis*	4 (7.7%)	4 (13.3%)	0.350
772	*772 base*	4 (7.7%)		0.173
609	*Penicillium corylphilum*	4 (7.7%)	2 (6.7%)	0.622
470	*470 base*	4 (7.7%)	4 (13.3%)	0.350
608	*Penicillium steckii*	4 (7.7%)	2 (6.7%)	0.622
655	*Trichoderma longibrachiatum*	3 (5.8%)		0.266
876	*Saccharomyces cerevisiae*	3 (5.8%)	6 (20.0%)	0.083
434	*Candida rugosa*	3 (5.8%)	6 (20.0%)	0.083
845	*Malassezia cuniculi*	3 (5.8%)	1 (3.3%)	0.544
558	*Candida tropicalis*	3 (5.8%)	2 (6.7%)	0.610
580	*Cladosporium cladosporioides*	3 (5.8%)		0.266
683	*Exophiala pisciphila*	3 (5.8%)		0.266
770	*770 base*	2 (3.8%)	3 (10.0%)	0.278
818	*818 base*	2 (3.8%)	1 (3.3%)	0.700
916	*916 base*	2 (3.8%)		0.411
970	*970 base*	2 (3.8%)		0.411
647	*Wickerhamomyces anomalus*	2 (3.8%)	5 (16.7%)	0.081
535	*535 base*	2 (3.8%)	1 (3.3%)	0.700
586	*Cryptococcus neoformans*	1 (1.9%)	1 (3.3%)	0.605
638	*Candida guilliermondii*	1 (1.9%)	3 (10.0%)	0.155
956	*956 base*	1 (1.9%)		0.639
677	*Exophiala lecanii-corni*	1 (1.9%)		0.639
547	*Trichosporon cutaneum*	1 (1.9%)		0.639
520	*520 base*	1 (1.9%)		0.639
487	*Dipodascus capitatus*	1 (1.9%)	1 (3.3%)	0.605
493	*Candida apicola*	1 (1.9%)	1 (3.3%)	0.605
540	*Candida krusei*	1 (1.9%)		0.639
750	*Candida kefyr*	1 (1.9%)		0.639

Only those detected as a peak with an area ≥1% of the total are listed with their origins.

*p<0.05 (Fisher’s exact test and Yates’ correction).

### Change in fungal populations following antifungal therapy

**Figure 3:**
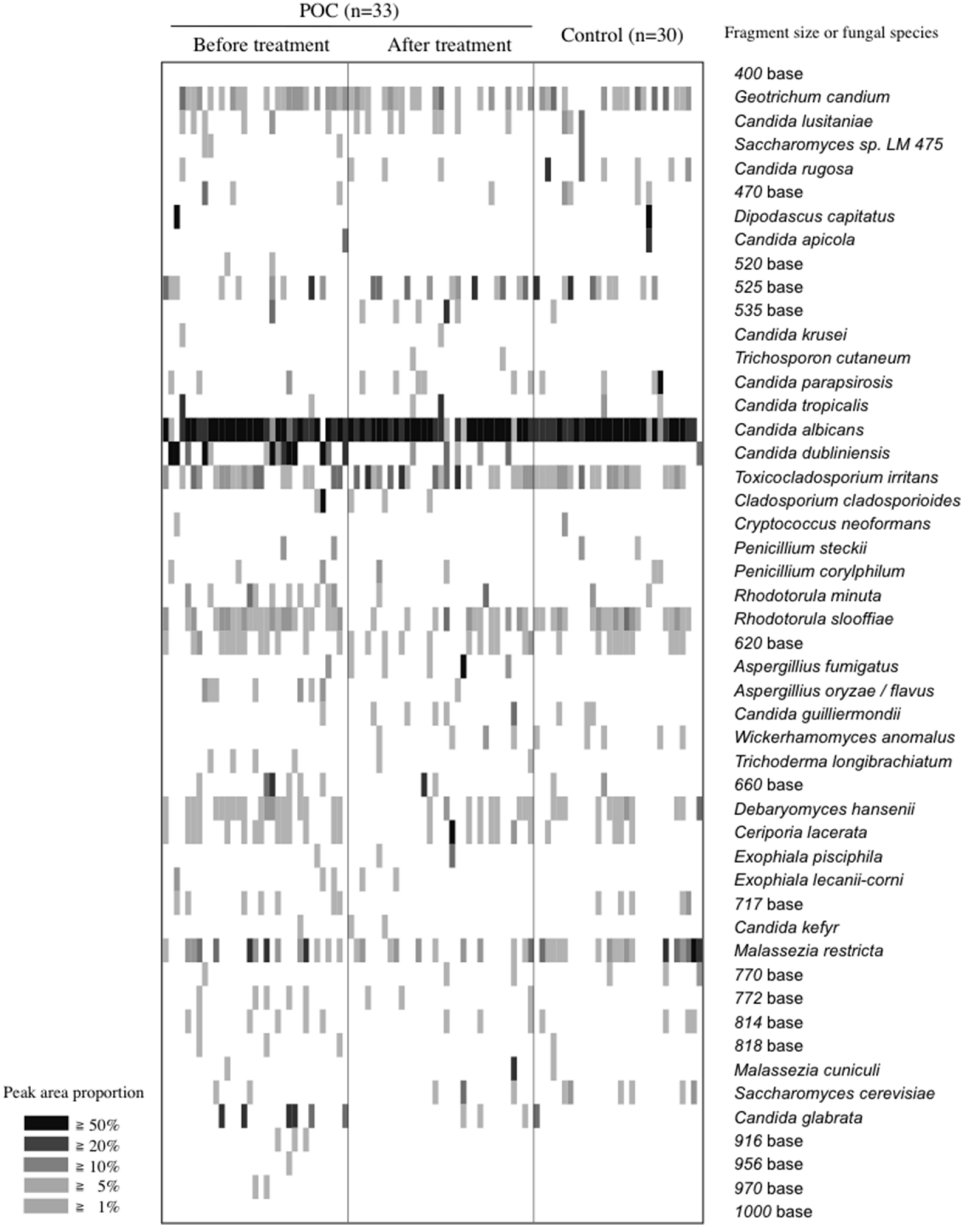
LH-PCR peak patterns from patients with POC before and after therapy. The peak area proportion (47 detected peaks) in each LH-PCR profile is represented by the gray-scale intensity of each grid.

**Figure 4:**
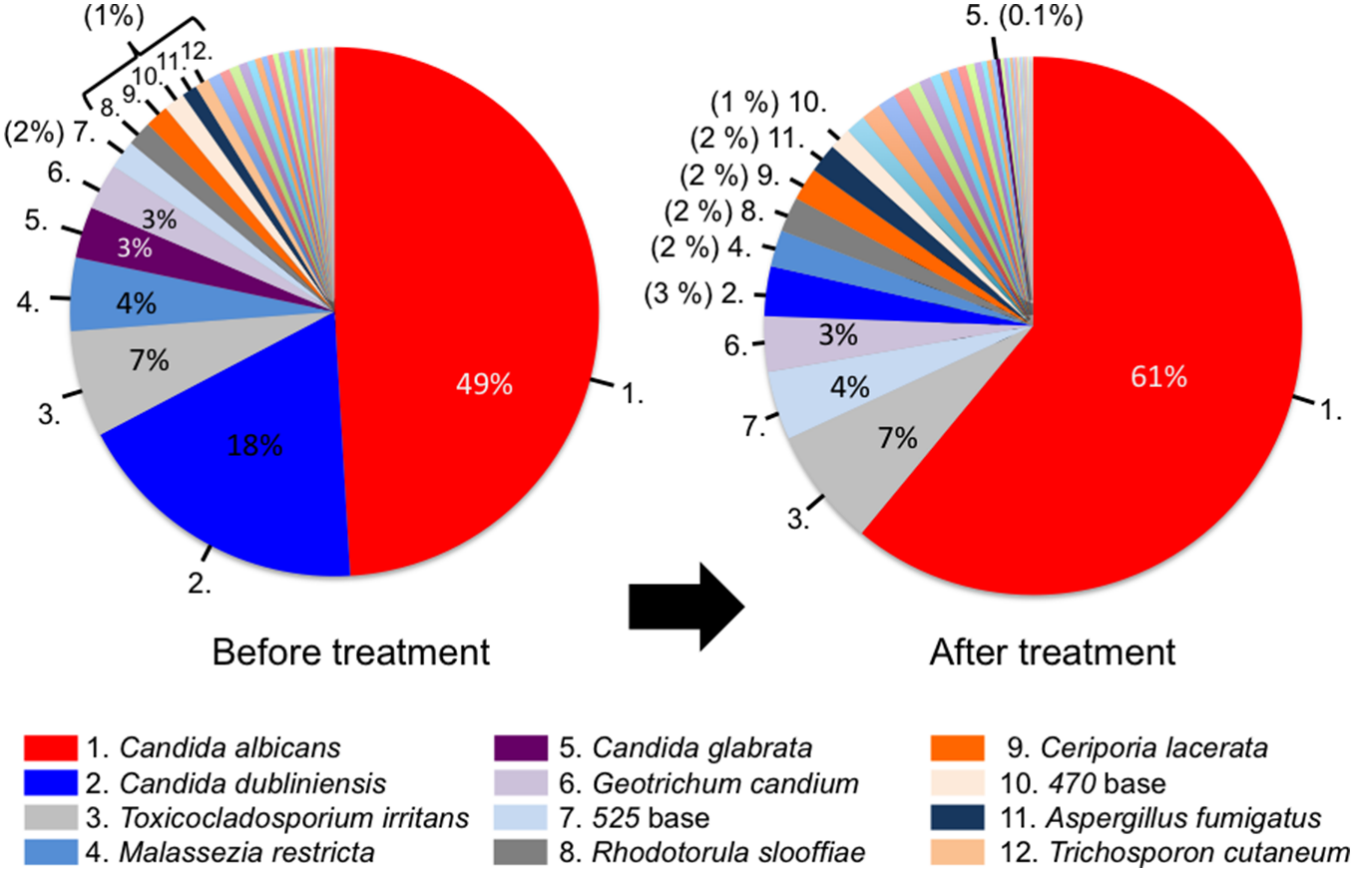
The mean composition ratio of fungal populations from patients with POC before and after therapy. Top 12 fungi of detection rate in POC before treatment group are shown.

Thirty-three of 52 patients with POC were available for serial assessments following treatment with the antifungal drug FLO-G. The average of treatment duration was The LH-PCR profiles are displayed as a gel-like image in Figure 3. After antifungal therapy, the detection ratio of *C. dubliniensis* was considerably decreased and the detection patterns in patients with POC were similar to those in controls. The total and average numbers of detection signals per person were significantly decreased (Table 2). In addition, the detection ratio of *C. albicans* increased, while that of *C. dubliniensis* almost disappeared (Figure 4).

### Relationships between treatment duration and clinical findings

**Figure 5:**
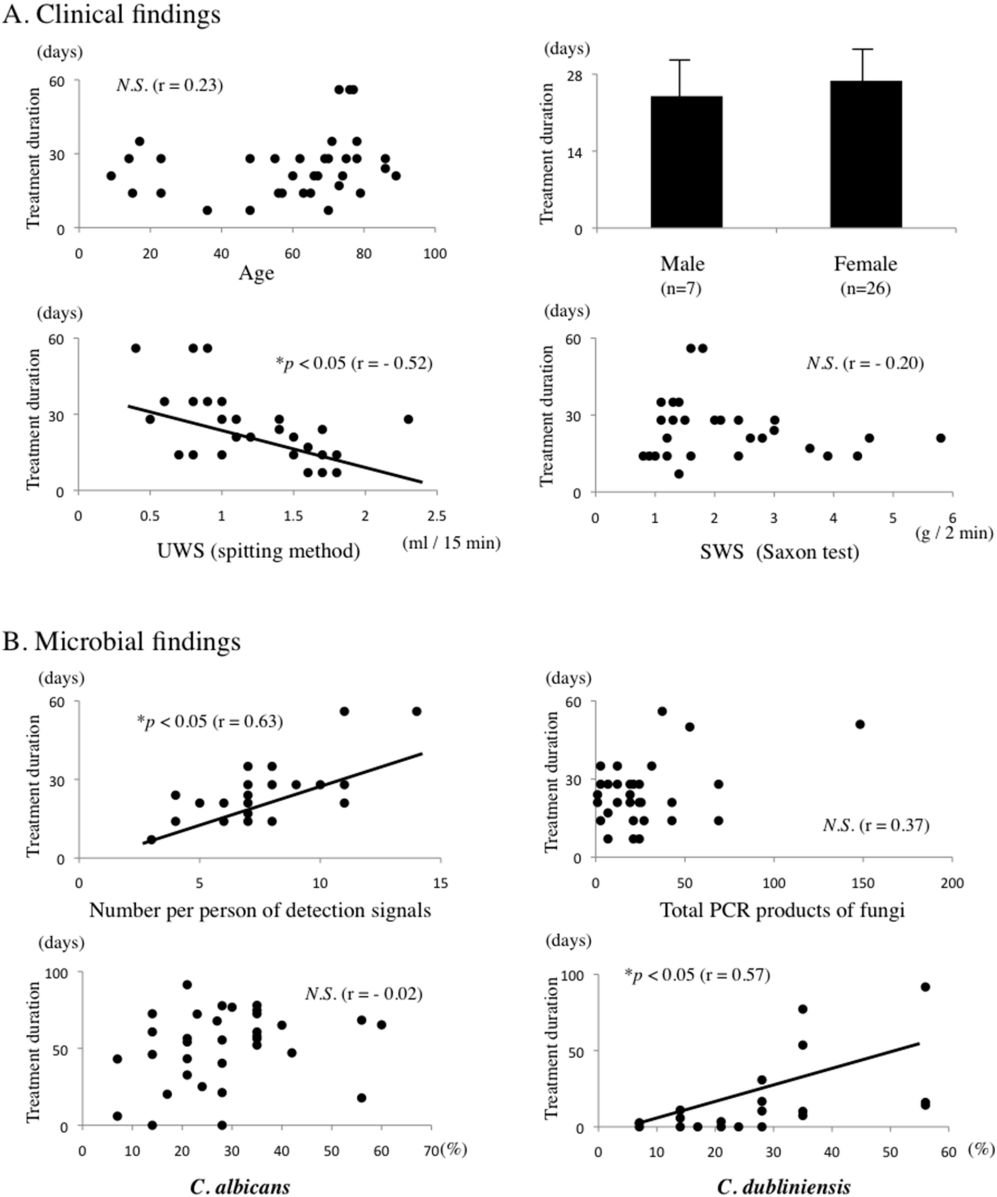
Correlation between treatment duration and clinical and microbial findings. A. Correlations between treatment duration and clinical findings including age, sex, unstimulated whole salivary flow rate (UWS), and stimulated whole salivary flow rate (SWS). B. Correlations between treatment duration and microbial findings, including number of detection signals, total PCR products, composition ratios of *C. albicans* and *C. dubliniensis*. Significance of differences between groups was determined by Student’s *t*-test and Spearman’s rank correlation (*p<0.05). *N.S.,* not significant.

To identify treatment-resistance factors in POC, we investigated the relationships between treatment duration and clinical findings in patients with POC. Treatment duration was negatively correlated with UWS, but there was no significant correlation between treatment duration and any other clinical findings including age, sex, and SWS (Figure 5A).

### Relationship between treatment duration and fungal populations

The relationships between treatment duration and fungal populations including total PCR products, number of detection signals per person and composition ratio of fungal populations were examined. Treatment duration was positively correlated with the number of detection signals per person and the composition ratio of *C. dubliniensis* (Figure 5B).

## Discussion

Indigenous bacteria or fungi in the oral cavity have been suggested as the cause of cerebrovascular disturbances [2], [18]. Fungal infection is generally caused by opportunistic pathogens mainly *Candida* species which are able to establish infections when the host’s immunocompetence is impaired. Such infections range from superficial infections of the skin and mucous membranes to life-threatening invasive infections of the blood and/or organs. An aging population together with advances in medical techniques such as graft surgery, immunosuppressive therapy and anticancer treatment, mean that the number of persons at risk of invasive mycoses continues to grow The numbers of infections caused by non-albicans *Candida* species or other fungi is also increasing. Moreover, some species have acquired resistance to antifungal agents. These results suggest changing patterns and trends in fungal flora [19]. Although candidiasis including POC is currently the major opportunistic infection of the oral cavity, an increase in the number of intractable cases has been reported suggesting an increase in diversity of the oral fungal flora with the resistant species. Traditionally, the fungal flora has been examined using cultivation-based methods and CHROMagar *Candida* can identify *C. albicans, C. parapsilosis, C. tropicalis, C. krusei* and *C. glabrata* on the basis of colony color. These are the most common *Candida* species but it is difficult to identify other less common fungal species using this method [19], [20]. LH-PCR analysis of the fungal ITS1-5.8S rRNA-ITS2 region has recently been developed allowing more exhaustive surveys of fungal communities including difficult-to-cultivate species. In this study, we evaluated the fungal flora from patients with POC using LH-PCR analysis of the fungal ITS1-5.8S rRNA-ITS2 region. The total PCR products and number of detected signals were significantly higher in patients with POC than in controls suggesting that the increases in numbers and diversity of the oral fungal flora may be involved in the pathogenesis of POC. In addition, the detection and composition ratio of *C. dubliniensis* were markedly higher in patients with POC.

*C. dubliniensis* was first isolated from HIV-infected individuals in 1995 prior to which the species could not be distinguished from *C. albicans* because of their phenotypic similarities [21], [22]. *C. dubliniensis* is commonly associated with oropharyngeal candidosis in HIV-infected and AIDS patients, and a high prevalence (15–30%) of *C. dubliniensis* in the oral cavities of HIV-infected and AIDS patients has been reported in several studies [23]. In contrast, only 3.5% of healthy individuals in Ireland were found to carry *C. dubliniensis* in the oral cavity. In the current LH-PCR analysis, the 569-base fragment corresponded to *C. albicans* while the 571- and 573-base fragments matched *C. dubliniensis*, which was rarely recognized in the healthy subjects. Although its infectivity and virulence remain unclear, *C. dubliniensis* might be a pathogenic fungus in POC. The results of gargle specimen in this study were consistent with those of tongue scraping samples (Figure S1).

To identify treatment-resistance factors, we examined the relationships between clinical findings (age, sex, SWS, UWS and lesion parts) and treatment duration. Only UWS was negatively correlated with the treatment duration. Human whole saliva contains several antibiotics such as lysozyme, lactoferrin, peroxidases, and salivary immunoglobulin A. Many studies have pointed to an association between oral candidiasis and constant hyposalivation [24], [25]. UWS reflects a constant moistness in the oral cavity and decreased UWS has been thought to increase the risk of *Candida* infection [26], [27]. Finally, we compared oral fungal flora (in terms of the mean number of detected signals, the total amount, and the composition ratio) and the treatment duration. The mean number of detected signals and the composition ratio of *C. dubliniensis* were positively correlated with the treatment duration. However, *C. dubliniensis* was commonly susceptible to almost all antifungal agents and showed low resistance. In contrast, *C. dubliniensis* isolated from HIV patients with recurrent oral candidiasis and receiving fluconazole therapy showed either low susceptibility or high resistance to fluconazole [28]. These results indicate that the diversity of the oral fungal flora and the component ratio of *C. dubliniensis* may make POC harder to treat. Identification of fungal populations using LH-PCR analysis may thus be useful for the early diagnosis and treatment of POC.

## Supporting Information

Figure S1 **The mean composition ratio of fungal populations in oral rinse and tongue scraping samples from patients with POC.** Top 12 fungi of detection rate in oral rinse group are shown.
(TIF)

## References

[1] Teramoto S, Fukuchi Y, Sasaki H, Sato K, Sekizawa K, et al (2008) High incidence of aspiration pneumonia in community- and hospital-acquired pneumonia in hospitalized patients: a multicenter, prospective study in Japan. Journal of the American Geriatrics Society 56: 577–579. 18315680 10.1111/j.1532-5415.2008.01597.x

[2] Lin HL, Chao CM, Lai CC (2013) The impact of Candida isolates on the outcome of aspiration pneumonia. American journal of infection control 41: 850–851. 10.1016/j.ajic.2013.04.021 23993763

[3] Krcmery V, Barnes AJ (2002) Non-albicans Candida spp. causing fungaemia: pathogenicity and antifungal resistance. The Journal of hospital infection 50: 243–260. 12014897 10.1053/jhin.2001.1151

[4] Jabra-Rizk MA, Torres SR, Rambob I, Meiller TF, Grossman LK, et al (2007) Prevalence of oral Candida species ina North American pediatric population. The Journal of clinical pediatric dentistry 31: 260–263. 19161062 10.17796/jcpd.31.4.820968206675v577

[5] Williams EJ, Embleton ND, Bythell M, Ward Platt MP, Berrington JE (2013) The changing profile of infant mortality from bacterial, viral and fungal infection over two decades. Acta paediatrica (Oslo, Norway: 1992) 102: 999–1004. 10.1111/apa.12341 23826761

[6] Ostrosky-Zeichner L (2012) Invasive mycoses: diagnostic challenges. The American journal of medicine 125: S14–24. 10.1016/j.amjmed.2011.10.008 22196205

[7] Le J, Tran TT, Bui I, Wang MK, Vo A, et al (2013) Time to initiation of antifungal therapy for neonatal candidiasis. Antimicrobial agents and chemotherapy 57: 2550–2555. 23507285 10.1128/AAC.02088-12 PMC3716169

[8] Eraso E, Moragues MD, Villar-Vidal M, Sahand IH, Gonzalez-Gomez N, et al (2006) Evaluation of the new chromogenic medium Candida ID 2 for isolation and identification of Candida albicans and other medically important Candida species. Journal of clinical microbiology 44: 3340–3345. 16954270 10.1128/JCM.00213-06 PMC1594741

[9] Yurayart C, Chindamporn A, Suradhat S, Tummaruk P, Kajiwara S, et al (2011) Comparative analysis of the frequency, distribution and population sizes of yeasts associated with canine seborrheic dermatitis and healthy skin. Veterinary microbiology 148: 356–362. 20961712 10.1016/j.vetmic.2010.09.020

[10] Ghannoum MA, Jurevic RJ, Mukherjee PK, Cui F, Sikaroodi M, et al (2010) Characterization of the oral fungal microbiome (mycobiome) in healthy individuals. PLoS pathogens 6: e1000713. 20072605 10.1371/journal.ppat.1000713 PMC2795202

[11] Li H, Takeshita T, Furuta M, Tomioka M, Shibata Y, et al (2012) Molecular characterization of fungal populations on the tongue dorsum of institutionalized elderly adults. Oral diseases 18: 771–777. 22642872 10.1111/j.1601-0825.2012.01944.x

[12] Kohler PF, Winter ME (1985) A quantitative test for xerostomia. The Saxon test, an oral equivalent of the Schirmer test. Arthritis and rheumatism 28: 1128–1132. 4052124 10.1002/art.1780281008

[13] Vitali C, Bombardieri S, Jonsson R, Moutsopoulos HM, Alexander EL, et al (2002) Classification criteria for Sjogren’s syndrome: a revised version of the European criteria proposed by the American-European Consensus Group. Annals of the rheumatic diseases 61: 554–558. 12006334 10.1136/ard.61.6.554 PMC1754137

[14] Takeshita T, Nakano Y, Yamashita Y (2007) Improved accuracy in terminal restriction fragment length polymorphism phylogenetic analysis using a novel internal size standard definition. Oral microbiology and immunology 22: 419–428. 17949346 10.1111/j.1399-302X.2007.00384.x

[15] Gardes M, Bruns TD (1993) ITS primers with enhanced specificity for basidiomycetes–application to the identification of mycorrhizae and rusts. Molecular ecology 2: 113–118. 8180733 10.1111/j.1365-294x.1993.tb00005.x

[16] Vilgalys R, Hester M (1990) Rapid genetic identification and mapping of enzymatically amplified ribosomal DNA from several Cryptococcus species. Journal of bacteriology 172: 4238–4246. 2376561 10.1128/jb.172.8.4238-4246.1990 PMC213247

[17] Liu Z (1996) Hetero-stagger cloning: efficient and rapid cloning of PCR products. Nucleic acids research 24: 2458–2459. 8710524 10.1093/nar/24.12.2458 PMC145923

[18] Pasqualotto AC (2009) Candida and the paediatric lung. Paediatric respiratory reviews 10: 186–191. 19879508 10.1016/j.prrv.2009.09.001

[19] Richardson MD (2005) Changing patterns and trends in systemic fungal infections. The Journal of antimicrobial chemotherapy 56 Suppl 1i5–i11. 16120635 10.1093/jac/dki218

[20] Arendrup MC (2013) Candida and candidaemia. Susceptibility and epidemiology. Danish medical journal 60: B4698. 24192246

[21] Sharifzadeh A, Khosravi AR, Shokri H, Asadi Jamnani F, Hajiabdolbaghi M, et al (2013) Oral microflora and their relation to risk factors in HIV+ patients with oropharyngeal candidiasis. Journal de mycologie medicale 23: 105–112. 23721997 10.1016/j.mycmed.2013.02.001

[22] Sullivan DJ, Westerneng TJ, Haynes KA, Bennett DE, Coleman DC (1995) Candida dubliniensis sp. nov.: phenotypic and molecular characterization of a novel species associated with oral candidosis in HIV-infected individuals. Microbiology (Reading, England) 141 (Pt 7): 1507–1521. 10.1099/13500872-141-7-1507 7551019

[23] Sullivan DJ, Moran GP, Coleman DC (2005) Candida dubliniensis: ten years on. FEMS microbiology letters 253: 9–17. 16213674 10.1016/j.femsle.2005.09.015

[24] Shinozaki S, Moriyama M, Hayashida JN, Tanaka A, Maehara T, et al (2012) Close association between oral Candida species and oral mucosal disorders in patients with xerostomia. Oral diseases 18: 667–672. 22548381 10.1111/j.1601-0825.2012.01923.x

[25] Bossola M, Tazza L (2012) Xerostomia in patients on chronic hemodialysis. Nature reviews Nephrology 8: 176–182. 22249779 10.1038/nrneph.2011.218

[26] Ihalin R, Loimaranta V, Tenovuo J (2006) Origin, structure, and biological activities of peroxidases in human saliva. Archives of biochemistry and biophysics 445: 261–268. 16111647 10.1016/j.abb.2005.07.004

[27] Klentrou P, Cieslak T, MacNeil M, Vintinner A, Plyley M (2002) Effect of moderate exercise on salivary immunoglobulin A and infection risk in humans. European journal of applied physiology 87: 153–158. 12070626 10.1007/s00421-002-0609-1

[28] Moran GP, Sullivan DJ, Henman MC, McCreary CE, Harrington BJ, et al (1997) Antifungal drug susceptibilities of oral Candida dubliniensis isolates from human immunodeficiency virus (HIV)-infected and non-HIV-infected subjects and generation of stable fluconazole-resistant derivatives in vitro. Antimicrobial agents and chemotherapy 41: 617–623. 9056003 10.1128/aac.41.3.617 PMC163761

