# Correction: Molecular Analysis of Fungal Populations in Patients with Oral Candidiasis Using Internal Transcribed Spacer Region

**DOI:** 10.1371/journal.pone.0110450

**Published:** 2014-10-03

**Authors:** The PLOS ONE Staff

The third author’s name is spelled incorrectly. The correct name is Toru Takeshita. The correct citation is: Ieda S, Moriyama M, Takeshita T, Maehara T, Imabayashi Y, et al. (2014) Molecular Analysis of Fungal Populations in Patients with Oral Candidiasis Using Internal Transcribed Spacer Region. PLoS ONE 9(6): e101156. doi:10.1371/journal.pone.0101156
