# Supplementary figures and images for: Molecular Analysis of Fungal Populations in Patients with Oral Candidiasis Using Internal Transcribed Spacer Region
Source: PLoS One. 2014 Jun 30;9(6):e101156. doi: 10.1371/journal.pone.0101156 (PMC4076276; doi:10.1371/journal.pone.0101156)

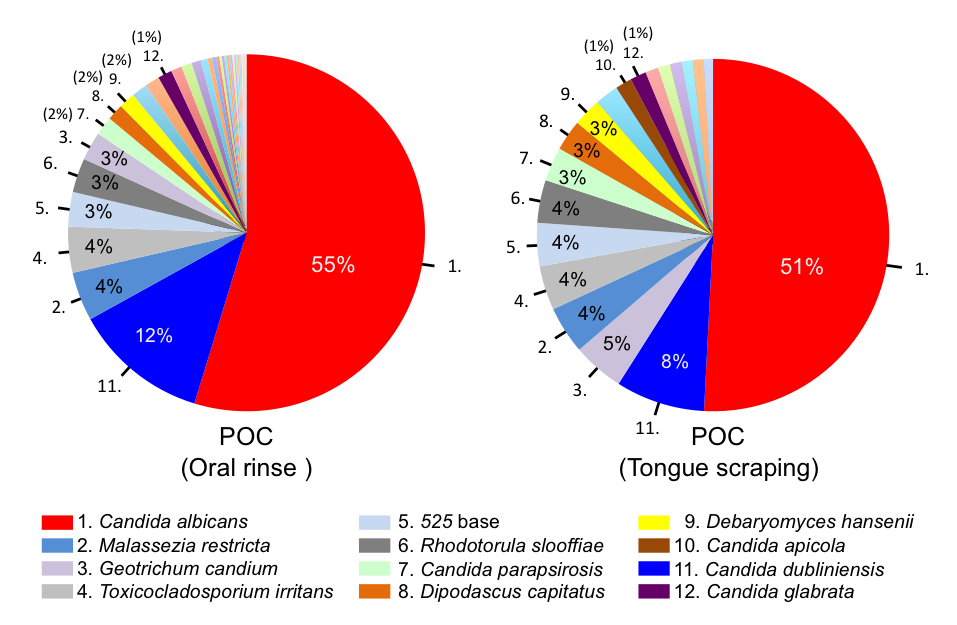

Supplement: Figure S1 — The mean composition ratio of fungal populations in oral rinse and tongue scraping samples from patients with POC. Top 12 fungi of detection rate in oral rinse group are shown. (TIF) [file pone.0101156.s001.tif]
